# Casein haplotype diversity in seven dairy goat breeds

**DOI:** 10.5194/aab-62-447-2019

**Published:** 2019-07-24

**Authors:** Andrea Criscione, Serena Tumino, Marcella Avondo, Donata Marletta, Salvatore Bordonaro

**Affiliations:** Department of Agriculture, Food and Environment, University of Catania, Catania, 95123, Italy

## Abstract

Selection, drift, gene flow and breeding have extensively shaped the genomic variability of domestic animals. In goat species, several mutations identified within the casein genes have been shown to affect the level of gene expression of milk production traits. The four casein genes – *CSN1S1*, *CSN2*, *CSN1S2* and *CSN3* – are organized in a cluster of 250 kb located in chromosome 6, and due to tight linkage, their genetic variability is well depicted by haplotypes which are transmitted to the progeny. Thirty single nucleotide polymorphisms (SNPs) located within the casein gene cluster were used to characterize the haplotype variability of six southern Italian goat breeds (Girgentana, Maltese, Rossa Mediterranea, Argentata dell'Etna, Messinese, Capra dell'Aspromonte). A representative sample of the Norwegian dairy goat breed (Norsk melkegeit) has been used as an out-group to obtain a weighted measure of genetic diversity in the metapopulation. A total of 54 haplotypes were detected among the seven breeds: 26, 9, 8 and 11 haplotypes were found at *CSN1S1*, *CSN2*, *CSN1S2* and *CSN3* respectively. The number of haplotypes per breed was 14 (Norwegian), 26 (Messinese), 27 (Rossa Mediterranea and Girgentana) and 31 (Maltese, Argentata dell'Etna and Capra dell'Aspromonte). The Maltese breed showed the highest number of private haplotypes, whereas the Norwegian goat recorded the highest number of shared haplotypes. The linkage disequilibrium analysis showed higher levels of association for the SNP pairs within casein loci than SNP pairs between casein loci, likely reflecting low levels of intra-genic recombination. The highest linkage disequilibrium values were found in *CSN1S1* and *CSN2* genes in all the breeds, except for Argentata dell'Etna and Rossa Mediterranea. The resolution of the haplotype diversity at the casein cluster can be exploited both for selective and conservative plans.

## 1 Introduction

The casein cluster is a genomic region of particular interest in dairy ruminants. In goat species, the genetic variability of caseins arises from several mutations that have been shown to affect gene expression level; the quantitative alleles modify the amount of single caseins in individual milk and, consequently, the technological and nutritional properties of goat milk (Marletta et al., 2007). Due to the tight association among the four casein genes, organized in a cluster of 250 kb, the study of the haplotype diversity, instead of single locus genotyping, seems to be preferable in view of planning selection strategies (Haplotype Assisted Selection) (Hayes et al., 2006; Caroli et al., 2006) and investigating breed genetic variability (Sacchi et al., 2005; Finocchiaro et al., 2008; Gigli et al., 2008; Küpper et al., 2010). South Italy preserves a rich heritage of dairy goat breeds: some of them originate from the Far and Middle East (Porter, 1996); others are derived from indigenous goats or were locally developed through different crossbreeding strategies. All these breeds have low population sizes; some of them are threatened by extinction and genetic erosion. In this regard, the molecular genetics can provide effective tools to effectively describe, monitor and manage genetic resources by handling variability within and among breeds. A recent study (Criscione et al., 2016) dealt with the genetic characterization of these goat genetic resources by means of a set of microsatellites (STRs), assumed to be selectively neutral markers and described the intra- and inter-breed diversity in view of planning conservation priorities. However, the genomic regions harbouring quantitative loci for milk, such as casein genes, are worth investigating in dairy breeds, in order to better analyse, characterize and explore their genetic diversity. The resolution of the haplotype diversity at the casein cluster can be exploited both for selective and conservative plans. The aim of this study was to investigate the haplotype diversity of the four casein genes' cluster in six southern Italian goat breeds (Argentata dell'Etna, Capra dell'Aspromonte, Girgentana, Maltese, Messinese, Rossa Mediterranea); a representative sample of the Norwegian dairy goat breed (Norsk melkegeit) was used as an out-group to obtain a weighted measure of genetic diversity in the metapopulation. The haplotype variability, assessed by 30 single nucleotide polymorphisms (SNPs), was used to estimate the genetic diversity within and between, as well as the genetic relationship among these dairy goat breeds. The use of these data for a potential casein haplotype assisted conservation plan was also briefly discussed.

## 2 Materials and methods

### 2.1 Sampling and SNPs analysis

A total of 207 goats and bucks representative of seven dairy goat populations were sampled in Italy (31 Girgentana GIR, 30 Argentata dell'Etna ARG, 30 Maltese MAL, 22 Rossa Mediterranea ROS, 30 Messinese MES and 31 Capra dell'Aspromonte ASP) and in Norway (33 Norwegian dairy goat NOR). All the animals were reared under real commercial farm conditions. Authorized personnel collected blood samples during the periodic veterinary control; therefore, no pain, suffering, distress or lasting harm was caused to the animals involved in the present study. The sampling was obtained from an average of nine herds per population to avoid closely related individuals, also according to the information obtained by farmers and genealogical data when available. More information on breeds' origin, sampling regions, total population size and the year of the studbook's establishment are reported in Criscione et al. (2016). Blood samples were collected in 10 mL vacutainer tubes (K3-EDTA). DNA was extracted from peripheral blood using the Illustrablood genomic Prep Mini Spin kit (GE Healthcare, Little Chalfont, UK). The whole sample was genotyped through a set of 30 SNP markers located in the promoter, exonic and intronic regions of the four casein genes (11 *CSN1S1*, 6 *CSN2*, 4 *CSN1S2* and 9 *CSN3*). The genetic characterization was performed using matrix-assisted laser desorption–ionization time-of-flight mass spectroscopy (MALDI-TOF MS) implemented in a MassARRAY System (Sequenom, San Diego, Ca, USA).

### 2.2 Statistical analysis

The software PHASE ver. 2.1, which implements a Bayesian statistical method, was used to reconstruct haplotypes from the allelic frequencies within each gene in each population (Stephens et al., 2001). The threshold frequency for the determination of haplotypes was set to 1 %.

The level of linkage disequilibrium (LD) between all pairs of loci in each breed was calculated through the software Haploview ver. 4.1 (Barrett et al., 2005) using the r2 statistic (Hudson, 1985).

The deviation from Hardy–Weinberg equilibrium was investigated, for each SNP in each population, by using the software Genepop ver. 4.2 (Raymond and Rousset, 1995) according to the procedure of a probability test.

The frequencies of the inferred haplotypes of the four casein genes in each goat breed were used to calculate the Nei's pairwise genetic distance ($D_{s}$) (Nei, 1972) by implementing the software Phylip 3.67 (Felsenstein, 2005). The Neighbour-Net algorithm was used to construct the phylogenetic network on $D_{s}$ distances by running the software SplitsTree4 ver. 4.14.2 (Huson and Briant, 2006). The haplotypes inferred at the four casein genes in each goat breed, were used also to implement the principal component analysis (PCA) by means of the software Minitab ver. 16.1 (Minitab, Inc., 2009)

## 3 Results

The results of the SNP genotyping are shown in Table 1. Three SNPs were found to be monomorphic in some breeds: SNP 13 in ROS, SNP 14 in GIR and NOR, SNP 19 in NOR. The distribution of the allelic frequencies was markedly different between the Norwegian sample and the Italian breeds: in particular 20 of 30 SNPs in NOR had a frequency of the minor allele (MAF) below 20 %, whereas a maximum of 7 of 30 SNPs reported the same frequency threshold among the Italian breeds (ASP). The lowest frequency of the minor allele (MAF) was 0.017 at SNP 13 and 14 in MAL. The deletion (allele D) at *CSN1S1* exon 9 (SNP 8) was found rarely in NOR (0.076), while it showed a frequency always above 30 % in all the Italian breeds and represented the major allele in MAL and ROS.

**Table 1:** SNP, gene, position, alleles (As), minor alleles (MAs)${}^{a}$, minor allele frequency (MAF) and p value of Hardy–Weinberg equilibrium test (HWp${}^{b}$) of the 30 casein SNP analysed in the seven goat breeds.

				ARG				ASP				GIR				MAL				MES				ROS				NOR		
SNP	Gene	Position	A	MA	MAF	HWp		MA	MAF	HWp		MA	MAF	HWp		MA	MAF	HWp		MA	MAF	HWp		MA	MAF	HWp		MA	MAF	HWp
1	*CSN1S1*	Promoter	A/G	A	0.467	**0.029**		G	0.468	0.075		G	0.371	*0.006*		G	0.383	**0.007**		A	0.383	0.055		G	0.432	**0.001**		A	0.197	1.000
2	*CSN1S1*	Promoter	A/G	A	0.433	1.000		A	0.387	0.444		A	0.500	**0.029**		G	0.383	0.703		A	0.450	0.481		G	0.409	0.071		A	0.197	1.000
3	*CSN1S1*	Promoter	A/G	G	0.417	1.000		G	0.387	1.000		G	0.355	**0.018**		G	0.300	0.688		A	0.467	0.724		G	0.295	1.000		A	0.197	1.000
4	*CSN1S1*	Promoter	C/T	C	0.417	1.000		C	0.387	1.000		C	0.355	**0.018**		C	0.300	0.688		T	0.467	0.724		C	0.295	1.000		T	0.197	1.000
5	*CSN1S1*	Promoter	A/G	G	0.417	1.000		G	0.387	1.000		G	0.355	**0.018**		G	0.300	0.688		A	0.467	0.724		G	0.295	1.000		A	0.197	1.000
6	*CSN1S1*	Exon 4	C/T	T	0.417	1.000		T	0.387	1.000		T	0.484	0.070		T	0.317	0.674		C	0.467	0.724		T	0.318	0.620		C	0.197	1.000
7	*CSN1S1*	Exon 4	C/G	C	0.433	0.718		C	0.387	1.000		C	0.500	**0.029**		C	0.317	0.674		G	0.467	0.724		C	0.318	0.620		G	0.197	1.000
8	*CSN1S1*	Exon 9${}^{c}$	C/D	D	0.400	1.000		D	0.339	1.000		D	0.419	0.277		C	0.417	0.706		D	0.317	0.674		C	0.432	0.419		D	0.076	1.000
9	*CSN1S1*	Intron 8	A/G	A	0.433	0.718		A	0.403	0.711		A	0.484	0.070		A	0.333	1.000		A	0.483	0.714		A	0.318	0.620		G	0.197	1.000
10	*CSN1S1*	Exon 10	C/G	C	0.433	0.718		C	0.403	0.711		C	0.484	0.070		C	0.333	1.000		C	0.483	0.714		C	0.318	0.620		G	0.197	1.000
11	*CSN1S1*	Exon 17	C/T	T	0.183	0.553		T	0.290	0.073		T	0.032	**0.016**		T	0.083	1.000		T	0.200	0.307		T	0.091	1.000		T	0.121	1.000
12	*CSN2*	Exon 7	C/T	C	0.167	0.568		C	0.290	0.378		C	0.032	**0.016**		C	0.067	1.000		C	0.150	1.000		C	0.091	1.000		C	0.091	1.000
13	*CSN2*	Promoter	A/G	G	0.067	1.000		G	0.081	1.000		G	0.032	**0.016**		G	0.017	–		G	0.050	1.000		A	0.000	–		G	0.076	1.000
14	*CSN2*	Promoter	A/G	A	0.050	1.000		A	0.032	1.000		G	0.000	–		A	0.017	–		A	0.033	1.000		A	0.045	1.000		G	0.000	–
15	*CSN2*	Promoter	A/G	G	0.450	0.152		G	0.306	0.217		G	0.484	0.070		A	0.400	0.447		G	0.350	0.418		A	0.432	0.419		G	0.076	1.000
16	*CSN2*	Promoter	A/T	A	0.483	0.160		A	0.339	1.000		T	0.323	**0.034**		T	0.350	0.418		A	0.383	0.703		T	0.341	0.338		A	0.076	1.000
17	*CSN2*	Promoter	C/T	C	0.450	0.476		T	0.371	0.449		C	0.290	0.071		C	0.333	0.682		T	0.400	1.000		C	0.341	0.338		T	0.076	1.000
18	*CSN1S2*	Exon 3	A/G	A	0.383	**0.009**		A	0.355	1.000		A	0.274	0.170		A	0.500	0.074		A	0.300	0.688		A	0.455	0.684		A	0.091	1.000
19	*CSN1S2*	Exon 16	C/G	C	0.433	**0.010**		C	0.403	**0.007**		G	0.484	0.279		G	0.300	0.217		G	0.317	**0.014**		G	0.386	0.653		C	0.000	–
20	*CSN1S2*	Exon 16	C/T	T	0.067	1.000		T	0.081	1.000		T	0.048	1.000		T	0.083	1.000		T	0.167	0.563		T	0.068	1.000		T	0.424	0.286
21	*CSN1S2*	Exon 16	A/T	T	0.167	1.000		T	0.242	0.327		T	0.065	1.000		T	0.233	1.000		T	0.333	0.423		T	0.227	0.538		T	0.424	0.286
22	*CSN3*	Promoter	A/G	A	0.250	0.646		A	0.194	0.307		A	0.242	**0.042**		A	0.300	1.000		A	0.300	0.688		A	0.250	0.568		A	0.333	1.000
23	*CSN3*	Promoter	A/T	T	0.250	0.646		T	0.242	1.000		T	0.274	0.170		T	0.300	1.000		T	0.317	0.674		T	0.250	0.568		T	0.379	0.722
24	*CSN3*	Promoter	A/T	A	0.250	0.646		A	0.194	0.307		A	0.226	0.145		A	0.300	1.000		A	0.300	0.688		A	0.250	0.568		A	0.333	1.000
25	*CSN3*	Promoter	C/G	C	0.250	0.646		C	0.242	1.000		C	0.274	0.170		C	0.300	1.000		C	0.317	0.674		C	0.250	0.568		C	0.379	0.722
26	*CSN3*	Promoter	G/T	G	0.250	0.646		G	0.194	0.307		G	0.226	0.145		G	0.300	1.000		G	0.300	0.688		G	0.250	0.568		G	0.333	1.000
27	*CSN3*	Promoter	G/T	G	0.200	0.559		G	0.177	1.000		G	0.242	**0.042**		G	0.267	0.383		G	0.283	1.000		G	0.227	0.271		G	0.333	1.000
28	*CSN3*	Promoter	C/T	T	0.433	0.069		T	0.435	0.157		C	0.210	**0.010**		C	0.450	0.157		C	0.383	0.055		C	0.432	0.103		C	0.424	0.286
29	*CSN3*	Promoter	G/T	T	0.433	0.718		T	0.419	1.000		T	0.145	0.093		T	0.300	0.378		T	0.233	0.632		T	0.273	0.623		T	0.424	0.286
30	*CSN3*	Promoter	A/G	G	0.267	1.000		G	0.274	0.179		A	0.435	0.469		G	0.350	1.000		G	0.400	0.264		G	0.432	1.000		G	0.197	0.307

${}^{a}$ The allele with the lowest frequency. ${}^{b}$ Significant p values are in bold. ${}^{c}$ Allele D at *CSN1S1* exon 9 stands for allele deletion.

A sizable number (13) of SNPs were in Hardy–Weinberg disequilibrium in GIR (Table 1), while a maximum of three SNPs were in HW disequilibrium in the other Italian breeds, and there were none in NOR. A total of 54 haplotypes were inferred within the four casein genes (Table 2): 26 at *CSN1S1*, 9 at *CSN2*, 8 at *CSN1S2* and 11 at *CSN3*. In each breed, the first two haplotypes always accounted for more than 50 % of the total frequency, ranging from 55 % to 84 % in the Italian breeds, except for MES and ROS at *CSN1S2*. NOR breed showed the highest frequency of the first two haplotypes at *CSN1S1*, *CSN2* and *CSN1S2*, always above 90 %. Private haplotypes had a frequency always below 5 % except for haplotype a16 in GIR (f=0.13). The breeds showing the highest number of haplotypes (31) were ARG, ASP and MAL (Table 3); the latter had also the highest number of private haplotypes. In contrast, NOR had the least number of haplotypes (14) together with the highest percentage of shared haplotypes.

**Table 2:** Inferred haplotypes per casein gene of the seven goat breed analysed (ARG, ASP, GIR, MAL, MES, ROS and NOR), haplotype code (Hc)${}^{*}$ and frequency (f) ≥ 1 %.

	ARG				ASP				GIR				MAL				MES				ROS				NOR		
	Haplotype	Hc	f		Haplotype	Hc	f		Haplotype	Hc	f		Haplotype	Hc	f		Haplotype	Hc	f		Haplotype	Hc	f		Haplotype	Hc	f
*CSN1S1*	GGGCGTCCACC	a1	0.33		GGGCGTCCACC	a1	0.33		GGGCGTCCACC	a1	0.32		AAATACGDGGC	a2	0.49		GGGCGTCCACC	a1	0.45		AAATACGDGGC	a2	0.35		GGGCGTCCACC	a1	0.80
	AAATACGDGGC	a2	0.24		AAATACGDGGC	a2	0.22		AAATACGDGGC	a2	0.31		GGGCGTCCACC	a1	0.26		AAATACGDGGC	a2	0.20		GGGCGTCCACC	a1	0.29		AAATACGCGGT	a5	0.12
	GAATACGDGGC	a3	0.09		AGATACGCGGT	a6	0.22		AGATATCCACC	**a16**	**0**.13		GGATACGCGGT	a7	0.04		GAATACGDGGC	a3	0.10		GAATACGDGGC	a3	0.17		AAATACGDGGC	a2	0.08
	GGGCGTCDACC	a4	0.07		GAATACGDGGC	a3	0.07		AAATACGCGGC	a17	0.10		GAATACGDGGC	a3	0.03		AAATACGCGGT	a5	0.09		GGATACGCGGT	a7	0.06				
	AAATACGCGGT	a5	0.07		AAATACGCGGT	a5	0.04		GGGCGTCDACC	a4	0.03		AGATACGDGGC	a14	0.02		GGGCGTCCGGT	a12	0.05		GGATACGDGGC	a21	0.04				
	AGATACGCGGT	a6	0.06		GGGCGTCDACC	a4	0.03		AAATACGCGGT	a5	0.03		AAATACGCGGC	a17	0.02		AAATACGCGGC	a17	0.03		GGATATCCACC	**a26**	0.02				
	GGATACGCGGT	a7	0.04		GGGCGTCCGGT	a12	0.02		GAATACGDGGC	a3	0.03		AGATACGCGGT	a6	0.01		GGGCGTCCACT	**a25**	0.03		AAATACGCGGT	a5	0.02				
	AGATACGCGGC	**a8**	0.03		GAATACGCACC	**a13**	0.02		AGATACGDGGC	a14	0.02		AAATACGCGGT	a5	0.01		GAATACGCGGT	a20	0.02		AAATACGCGGC	a17	0.01				
	GGGCGCCCACC	**a9**	0.02		AGATACGDGGC	a14	0.02		AAATACCDGGC	**a18**	0.01		AGGCGTCCACC	**a19**	0.01												
	GAATACGCGGC	**a10**	0.01		AAATACGCACC	**a15**	0.02						GAATACGCGGT	a20	0.01												
	AAATATCCACC	**a11**	0.01										GGATACGDGGC	a21	0.01												
													AAGCGTCCACC	**a22**	0.01												
													GAATACGDACC	**a23**	0.01												
													GAGCGTCCACC	**a24**	0.01												
*CSN2*	TAGGAT	b1	0.44		TAGATC	b2	0.34		TAGGAT	b1	0.48		TAGGAT	b1	0.58		TAGATC	b2	0.46		TAGGAT	b1	0.56		TAGATC	b2	0.83
	TAGATC	b2	0.30		TAGGAT	b1	0.30		TAGATC	b2	0.26		TAGATC	b2	0.26		TAGGAT	b1	0.34		TAGATC	b2	0.26		CGGATC	b4	0.09
	TAGATT	b3	0.07		CAGATC	b6	0.18		TAGAAT	b7	0.19		TAGAAT	b7	0.06		CAGATC	b6	0.07		TAGAAT	b7	0.09		TAGGAT	b1	0.08
	CGGATC	b4	0.07		CGGATC	b4	0.08		TAGATT	b3	0.03		CAGATC	b6	0.03		CGGATC	b4	0.05		CAAATC	b5	0.04				
	CAAATC	b5	0.05		CAAATC	b5	0.03		CGGATC	b4	0.03		CAAATC	b5	0.02		TAGAAT	b7	0.03		CAGATC	b6	0.04				
	CAGATC	b6	0.04		TAGAAT	b7	0.03						TAGGTT	**b9**	0.02		CAAATC	b5	0.02		CAGGAT	b8	0.01				
	TAGAAT	b7	0.03		TAGATT	b3	0.03																				
	CAGGAT	b8	0.01																								
*CSN1S2*	GGCA	c1	0.37		GGCA	c1	0.33		GGCA	c1	0.35		ACCA	c3	0.49		GGCA	c1	0.22		AGCA	c2	0.25		GCCA	c5	0.50
	AGCA	c2	0.22		AGCA	c2	0.26		GCCA	c5	0.32		GCCA	c5	0.16		ACCA	c3	0.21		ACCA	c3	0.20		GCTT	c6	0.40
	ACCA	c3	0.16		GCCT	c4	0.15		AGCA	c2	0.14		GCCT	c4	0.15		GCCT	c4	0.16		GCCT	c4	0.18		ACCA	c3	0.07
	GCCT	c4	0.11		ACCA	c3	0.10		ACCA	c3	0.12		GGCA	c1	0.10		GCCA	c5	0.16		GCCA	c5	0.16		ACTT	**c8**	0.02
	GCCA	c5	0.08		GCCA	c5	0.06		GCTT	c6	0.05		GCTT	c6	0.08		GCTT	c6	0.16		GGCA	c1	0.16				
	GCTT	c6	0.05		GCTT	c6	0.06		GCCT	c4	0.01						AGCA	c2	0.08		GCTT	c6	0.04				
					GGCT	**c7**	0.04																				
*CSN3*	GATGTTCTA	d1	0.43		GATGTTCTA	d1	0.41		GATGTTTGG	d3	0.52		GATGTTCTA	d1	0.30		GATGTTTGG	d3	0.30		GATGTTTGG	d3	0.31		GATGTTCTA	d1	0.42
	ATACGGTGA	d2	0.20		GATGTTTGG	d3	0.19		ATACGGTGA	d2	0.22		ATACGGTGA	d2	0.27		ATACGGTGA	d2	0.28		GATGTTCTA	d1	0.27		ATACGGTGA	d2	0.33
	GATGTTTGG	d3	0.18		ATACGGTGA	d2	0.18		GATGTTCTA	d1	0.14		GATGTTTGG	d3	0.25		GATGTTCTA	d1	0.23		ATACGGTGA	d2	0.20		GATGTTTGG	d3	0.20
	GATGTTCGA	d4	0.10		GATGTTCGA	d4	0.09		GATGTTCGG	d6	0.03		GATGTTCGA	d4	0.08		GATGTTCGG	d6	0.10		GATGTTCGA	d4	0.09		GTTCTTTGA	d7	0.05
	ATACGTTGG	d5	0.05		GATGTTCGG	d6	0.06		GATGTTCGA	d4	0.03		GATGTTCGG	d6	0.07		GATGTTCGA	d4	0.05		GATGTTCGG	d6	0.07				
	GATGTTCGG	d6	0.03		GTTCTTTGA	d7	0.05		GTTCTTTGG	**d8**	0.03		ATACGTTGG	d5	0.03		ATACGTTGA	**d10**	0.02		ATACGTTGG	d5	0.03				
					ATACGTTGG	d5	0.02		ATTCTGTGA	**d9**	0.01										ATACGGTGG	**d11**	0.02				

${}^{*}$ The codes of private haplotypes are highlighted in bold type.

**Table 3:** Number of inferred haplotypes (f≥1 %) per breed and gene, percentage of private haplotypes (% PH), and percentage of haplotypes shared among all seven goat breeds (% SH7).

Breed	*CSN1S1*	*CSN2*	*CSN1S2*	*CSN3*	tot	% PH	% SH7
ARG	11	8	6	6	31	12.9	35.5
ASP	10	7	7	7	31	9.7	35.5
GIR	9	5	6	7	27	14.8	40.7
MAL	14	6	5	6	31	16.1	35.5
MES	8	6	6	6	26	7.7	42.3
ROS	8	6	6	7	27	7.4	40.7
NOR	3	3	4	4	14	7.1	78.6

In Table 4 are reported the results of LD analysis as average values within each locus and across loci in each breed. In all the breeds, the intra-locus LD level highlighted the highest values at *CSN1S1* and then at *CSN3*, *CSN2* and *CSN1S2*, in order of decreasing SNP number. NOR showed the highest level of LD within each of the casein genes, with a very high value at *CSN1S1*. Across the genes, very high LD values were found between *CSN1S1* and *CSN2*, in all the breeds except for ARG and ROS.

**Table 4:** Average linkage disequilibrium within and between casein loci per breed.

Breed		*CSN1S1*	*CSN2*	*CSN1S2*	*CSN3*
ARG	*CSN1S1*	0.571	0.031	0.003	0.007
	*CSN2*		0.260	0.025	0.027
	*CSN1S2*			0.147	0.055
	*CSN3*				0.528
ASP	*CSN1S1*	0.556	0.103	0.023	0.027
	*CSN2*		0.240	0.027	0.015
	*CSN1S2*			0.153	0.026
	*CSN3*				0.475
GIR	*CSN1S1*	0.544	0.209	0.018	0.077
	*CSN2*		0.305	0.014	0.027
	*CSN1S2*			0.149	0.046
	*CSN3*				0.483
MAL	*CSN1S1*	0.631	0.178	0.061	0.012
	*CSN2*		0.227	0.039	0.037
	*CSN1S2*			0.149	0.041
	*CSN3*				0.528
MES	*CSN1S1*	0.636	0.101	0.026	0.012
	*CSN2*		0.239	0.019	0.034
	*CSN1S2*			0.171	0.018
	*CSN3*				0.516
ROS	*CSN1S1*	0.617	0.051	0.047	0.046
	*CSN2*		0.360	0.053	0.012
	*CSN1S2*			0.142	0.046
	*CSN3*				0.506
NOR	*CSN1S1*	0.801	0.339	0.011	0.078
	*CSN2*		0.387	0.024	0.085
	*CSN1S2*			0.383	0.030
	*CSN3*				0.568

**Figure 1:**
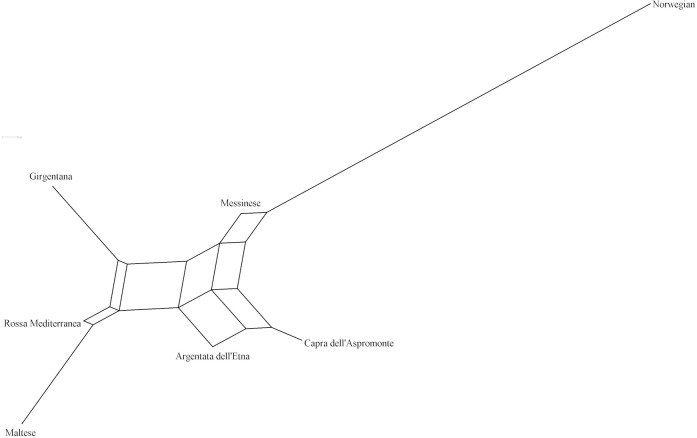
Neighbour-Net obtained from $D_{s}$ distance among the seven goat breeds.

**Figure 2:**
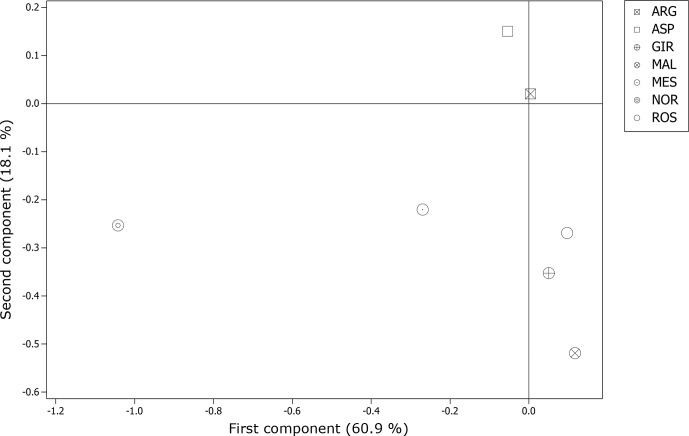
Score plot of the principal component analysis on inferred haplotypes among the seven goat breeds.

The matrix of pairwise $D_{s}$ genetic distance (Table 5) reported NOR and MES as the most and the least distinctive breed respectively. ASP and ARG highlighted the lowest pairwise value of the matrix, while NOR and MAL were the highest in the data set. Among the Italian goats, GIR and MAL were the most genetically distant breeds, even if the pairwise values of $D_{s}$ distance among the Italian goat populations were mostly comparable. The representation of the $D_{s}$ genetic distance using the Neighbour-Net algorithm (Fig. 1) clearly shows the dichotomy between the Norwegian goats and the Italian breeds, in which the three most selected breeds (GIR, ROS and MAL) grouped in a cluster separated from the mountain populations (MES, ASP and ARG). The haplotype frequencies, condensed in the principal component analysis (PCA), showed a spatial distribution of goat breeds (Fig. 2) in which the NOR was clearly separated from the Italian goats, the most selected Sicilian breeds (GIR, MAL and ROS) were clustered closely and were separated from MES and from the ARG-ASP group.

**Table 5:** Pairwise Nei's genetic distance ($D_{s}$) between the seven goat breeds.

	ARG	ASP	GIR	MAL	MES	ROS	NOR
ARG	–	0.046	0.179	0.177	0.112	0.088	0.437
ASP		–	0.250	0.275	0.132	0.152	0.410
GIR			–	0.209	0.151	0.119	0.492
MAL				–	0.161	0.089	0.573
MES					–	0.113	0.194
ROS						–	0.531
NOR							–
Mean	0.173	0.211	0.233	0.247	0.144	0.182	0.439

## 4 Discussion

The demographic history of a breed, including gene flow, drift and bottleneck events, shapes the distribution of nucleotide polymorphisms and the extent of genomic linkage disequilibrium (Nordborg and Tavare, 2002); therefore, the estimate of the haplotype variation and the LD in a casein cluster are very informative in estimating the effects of the selection, migrations or admixture in dairy goat populations.

Among the six Italian breeds, a rather high number of haplotypes per population was observed (from 26 in MES to 31 in ARG, ASP and MAL) with a frequency distribution and a number of private haplotypes, which denoted a high rate of within-breed variability and, at least partly, the absence of systematic breeding strategies. In contrast, the NOR breed showed the least within-breed genetic variability; the low values of the MAF for most of the 30 SNPs, the low number of haplotypes per locus and the unique private haplotype c8 (*CSN1S2* locus) described a very low variability in the casein cluster as well as strong distinctiveness. These findings could be linked to the selection management and the low number of founders of the Norwegian goat breed (Hayes et al., 2006). In fact, the planned mating system that uses buck circles on the national territory has undergone a downsizing following the implementation of the national programme for the eradication of caprine arthritis encephalitis virus since 2001 (Ådnøy, 2014); the number of available Norwegian bucks has decreased, and this has probably reduced genetic diversity. Nevertheless, the marked homogeneity of the casein cluster highlighted here does not seem to have led to a severe inbreeding rate, as shown by the population study through anonymous neutral marker STRs (Criscione et al., 2016).

In the Italian breeds the percentage of shared haplotypes (never >42.3 %) was quite low in comparison with NOR (78.6 %). These data are partially unexpected taking into account the gene flow that can potentially occur between Italian populations reared in the same area, in extensive systems and in the absence of structured selective breeding programmes, but they are in agreement with the pronounced degree of biodiversity and morphological differentiation described in these local breeds. In particular, MAL, with its high number of haplotypes and private combinations, seems to retain a great amount of heterogeneity mainly located in the *CSN1S1* locus. It is also worth noting the presence of a peculiar allelic distribution on SNP 8, in which the deletion (exon 9) represents the major allele in MAL and ROS goats. This mutation has previously been described as common in some Mediterranean breeds, including GIR and MAL (Sacchi et al., 2005; Gigli et al., 2008; Finocchiaro et al., 2008).

The level of linkage disequilibrium for pairs of markers within each casein locus was higher than for pairs of markers from different loci, as reported by Hayes et al. (2006) and Finocchiaro et al. (2008). The highest LD values were found in *CSN1S1* and *CSN2* genes in all breeds, except for ARG and ROS (Table 4).

The genetic relationship among the breeds, described according to $D_{s}$ distance, showed the phylogenetic distinctness of NOR from the Italian goat breeds. The set of SNPs used for the genetic characterization separated clearly the most selected dairy breeds GIR, MAL and ROS from the unselected populations (MESS, ASP and ARG) reared in mountainous regions and generally less productive. The spatial distribution of the seven goat breeds, represented through the score plot of the first two components of the PCA, was highly comparable to that of the Neighbour-Net built on the $D_{s}$ distance. Overall, 79 % of the variance showed two main results, as expected: the high distance between the NOR and the Italian goats as well as the clustering of the Sicilian breeds, which have been historically subjected to selection for dairy purposes (GIR, MAL and ROS). This last outcome slightly differs from the previous study, carried out by means of 20 microsatellites, which highlighted a clear admixture between ROS and the genetic pool of the mountainous population (Criscione et al., 2016), and it is due primarily to the use of different unlinked and non-neutral markers, which showed a different pattern of evolution.

Instead, given the remarkable genetic and geographical separation of NOR from the Italian breeds, the high level of shared haplotypes (78 %) between the Norwegian sample and the Italian breeds seems quite surprising. Due to the great geographical distance and the reproductive isolation, an explanation is not straightforward. Our findings confirm the low haplotypic variability at the casein cluster detected by Finocchiaro et al. (2008) in a different sample of GIR and NOR goats and the general assessment that goat populations show a low genetic differentiation rate (Luikart et al., 2001; Canon et al., 2006; Nicoloso et al., 2015). The severe shortage of haplotypes in the NOR breed also reflects the high geographical distance from the centre of goat domestication.

In the light of the casein haplotype diversity and according to the relatively rich patrimony of rare private haplotypes, all the Italian breeds are worth safeguarding. The endangered GIR, ASP and especially MAL breeds seem to acquire conservation priorities in the context of the southern Italian dairy goat, thanks to their degree of distinction and the private allelic combinations they have maintained.

## 5 Conclusion

Understanding the extent, distribution and origin of current genetic diversity in livestock populations requires diverse sources of information, including molecular data from different classes of markers. This study analysed an important region in the goat genome: the casein cluster located in chromosome 6. The results presented here have been compared with those obtained from the characterization of the same sampling by selectively neutral marker STRs. The characterization by means of this set of 30 SNPs highlighted a significant genetic diversity and differentiation among the six dairy goat breeds reared in southern Italy. All the Italian breeds, but mainly Maltese and to some extent Girgentana and Capra dell'Aspromonte, showed a relevant rate of genetic distinctness, especially when compared with the Norwegian breed. All these Italian breeds are reared in traditional management systems and in marginal areas, and some of them are endangered. Our findings could help interpret the evolutionary history of these breeds and represent a potential tool for the development of future management, conservation and breeding strategies.

## Data Availability

The data set is available upon request from the corresponding author.
